# Turtle Herbivory Suppresses Seagrass Function but Reveals Recovery Potential in a Remote Coral Reef Atoll

**DOI:** 10.1002/ece3.74466

**Published:** 2026-10-24

**Authors:** Caitlyn O'Dea, Kathryn McMahon, Osman Macrae, Shakirin Keegan, Sahira Bell

**Affiliations:** ^1^ Centre for Marine Ecosystems Research, School of Science Edith Cowan University Joondalup Western Australia Australia; ^2^ Centre for People Place and Planet Edith Cowan University Joondalup Western Australia Australia; ^3^ Cocos Marine Care Incorporated Home Island Cocos (Keeling) Islands; ^4^ Sea Country Solutions Pty Ltd Margaret River Western Australia Australia; ^5^ Shire of Cocos (Keeling) Islands Home Island Cocos (Keeling) Islands

## Abstract

1. Seagrasses are recognised as ecologically and culturally significant habitats across the globe supporting human livelihoods, biodiversity, and carbon storage. In the Cocos (Keeling) Islands, a remote coral reef atoll in the eastern Indian Ocean, seagrasses provide ecosystem services and support culturally, ecologically, and economically important species. However, these meadows have suffered devastating declines, with ongoing green turtle herbivory identified as a key pressure restricting *Thalassia hemprichii* persistence.
2. To examine how herbivory shapes seagrass traits and function, we compared plots protected from grazing for over two years using exclusion cages against continuously grazed plots across two sites.
3. Seagrass traits at the meadow‐scale (shoot density, leaf area and biomass), as well as some functionality (primary production and nutrient storage capacity), were improved with protection from herbivory pressure. Substantial reductions in the belowground biomass in the grazed plots indicate that these seagrasses are operating under severe metabolic constraints. In contrast, some plant‐scale traits (photosynthetic rate and growth rate) were not altered despite ongoing herbivory pressure, indicating some capacity for recovery at the individual plant scale.
4. Representing one of the longest turtle exclusion studies in seagrass ecosystems, these results demonstrate that herbivory is driving systemic degradation across multiple biological scales, but these seagrasses currently retain the capacity to recover once grazing pressure is reduced.
5. *Synthesis*. Tropical seagrass meadows subjected to continuous herbivory pressure can persist in a chronically depleted state. Herbivore exclusion experiments demonstrate that reducing herbivory can unlock the recovery potential of seagrass. These findings highlight the need for nuanced management strategies to safeguard these meadows in a time where seagrasses continue to decline at both localised and global scales, and the global turtle populations are simultaneously on the rise.

## Graphical abstract

Continuous herbivory pressure can drive degradation across multiple biological scales, but herbivore exclusion shows that seagrasses at the Cocos (Keeling) Islands currently retain the capacity to recover.

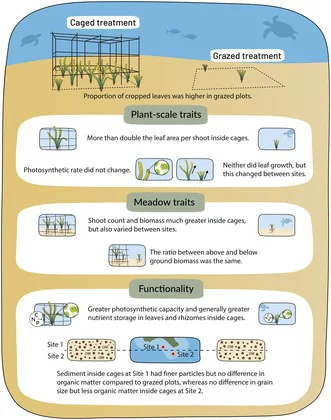

## 1 Introduction

Herbivory is a fundamental process that occurs across the planet in almost every environment. Plant and animal interactions have coevolved over millennia, (Huntly 1991; Estes et al. 2011; Bakker, Pagès, et al. 2016; Steneck et al. 2017) playing an important role in shaping the structure and function in ecosystems (Engelhardt and Ritchie 2001; Kindinger et al. 2024; Trepel et al. 2024). Like their terrestrial counterparts, aquatic herbivores can exert top‐down control on submerged aquatic vegetation that influences abundance, productivity, species composition and diversity with positive, negative or mixed consequences (Strauss and Agrawal 1999; Heck and Valentine 2007; Bakker, Wood, et al. 2016). Imbalances in plant‐herbivore relationships are possible when, for instance, there is a mismatch between the food requirements and availability in an ecosystem (Valentine and Heck 2021).

Green turtles ( *Chelonia mydas* ) have suffered declines across the twentieth century due to climate change, habitat loss and overharvesting (Hays et al. 2025). Following decades of extensive conservation efforts, this species population trend is generally increasing or stable across its global range (Hays et al. 2025). However, the role of this herbivore as ecosystem engineers inevitably reasserts itself along with cascading consequences for the seagrass meadows that sustain them (Christianen et al. 2021). Increased turtle populations represent increased herbivory pressure on seagrasses, and impacts have been reported across tropical seagrass ecosystems in Indonesia (Christianen et al. 2014), India (Kelkar et al. 2013; Gangal et al. 2021), Bermuda (Fourqurean et al. 2019), the Caribbean (Gulick et al. 2020; Christianen et al. 2023), the United States of America (Rodriguez and Heck 2021), and Australia (Heithaus et al. 2014; Buckee et al. 2021). The return of green turtles to their historical role is presenting new challenges for the concurrent conservation of both turtles and their foraging habitat upon which they depend (Christianen et al. 2021).

Herbivory is a natural process which can stimulate productivity, flowering and seed dispersal (Strauss and Agrawal 1999; Vergés et al. 2008; Gulick, Johnson, et al. 2021). However, intensive herbivory can decrease productivity and performance of individual plants (Fourqurean et al. 2010), eventually causing meadow‐scale changes in abundance and subsequently the critical ecological functions they perform (Fourqurean et al. 2019; Christianen et al. 2021; Gangal et al. 2021). Some ecological functions, such as habitat provision (Bell and Westoby 1986; Gangal et al. 2021), sediment trapping capacity (Johnson et al. 2019; James et al. 2020) and nutrient uptake (Moran and Bjorndal 2005; Aoki et al. 2020) are intrinsically linked to traits of individual plants and the broader meadow (Scott et al. 2018; Christianen et al. 2021) and may be impaired under intensive herbivory pressure. In some cases, seagrasses have declined to the point of ecological collapse where the largely unvegetated habitat represents an alternative stable state where functionality is absent and recovery is unlikely without intervention (Fourqurean et al. 2019; Buckee et al. 2021). When seagrass can persist despite constant herbivory pressure, it is possible that the condition can be restored, as well as their important ecological functions. However, few studies have investigated the response beyond a year of protection (Putillo‐Wehry et al. 2025) There is an urgent need to understand the realised impact of turtle grazing on seagrass habitats and the ability of meadows to provide critical ecosystem services (Reynolds et al. 2016).

Like many other seagrass ecosystems worldwide, the meadows at the Cocos (Keeling) Islands (CKI) have suffered widespread declines hypothesised to be due to a combination of turbidity associated with coastal development, episodic marine heat waves, and persistent herbivory by local turtle populations (Buckee et al. 2021; Konzewitsch and Evans 2024). Community members at the CKI were concerned that intensive turtle grazing was preventing seagrass recovery despite seagrass remaining persistent across the historical extent of the meadows in the lagoon. Subsequent lagoon‐wide surveys completed in collaboration with the community confirmed that seagrass remained persistent across much of the historical distribution, albeit at extremely low cover (Bell et al. 2025). A two‐year herbivore exclusion experiment further demonstrated that turtle herbivory is currently a key driver maintaining these meadows at extremely low cover (Bell et al. 2025, Bell et al. unpublished manuscript).

While this previous work established the overarching role of herbivory in shaping seagrass condition and recovery trajectories, it remains unclear if seagrass exposed to intensive herbivory retains the capacity to recover important traits and ecosystem functions once grazing pressure is reduced. Recovery in this context is defined as the reestablishment of structure and function once disturbance is relieved (Unsworth et al. 2015; O'Brien et al. 2018). Because grazing frequently involves the direct removal of aboveground tissues (Bjorndal 1980; Gulick, Meylan, et al. 2021), protection from herbivory would be expected to increase leaf biomass and leaf area. Less certain is whether a release from grazing also alters photosynthesis or nutrient content. A recent meta‐analysis by Putillo‐Wehry et al. (2025) identifies one study reporting no difference in the photosynthetic response of seagrass to turtle herbivory (Christianen et al. 2012). Nutrient content can increase or be unaffected following grazing (Moran and Bjorndal 2005; Fourqurean et al. 2010; Christianen et al. 2012; Molina Hernández and van Tussenbroek 2014; Lacey et al. 2014; Holzer and McGlathery 2016; Scott et al. 2020), however, this has not been scaled to storage capacity. In this study, we assess the effects of long‐term protection from herbivory on seagrass plant‐scale and meadow traits, as well as functional attributes linked with primary productivity, nutrient storage, and sediment processes. We compared seagrass protected from herbivory within exclusion cages for more than two years with adjacent seagrass exposed to natural grazing pressure. We hypothesise that the exclusion of turtles would result in reductions in traits and functions within cages compared to plants exposed to herbivory. Resolving these responses is necessary to interpret the condition of meadows that support herbivore populations and to inform the concurrent management of turtles and the habitats that sustain them.

## 2 Materials and Methods

### 2.1 Study Site

This experiment was conducted in the Cocos (Keeling) Islands (CKI); a remote tropical coral reef atoll situated in the Indian Ocean, approximately 2100 km north‐west of mainland Australia (Figure 2). The extensive coral reef and lagoon habitats support diverse marine species and livelihoods of the ~600 residents, most of whom are Cocos Malay and reside on Home Island (*Pulu Kampong*) (Herriman 2022; Weeks et al. 2024).

Seagrass, known as ‘*lumut*’ in the Cocos Malay language, is important to the local community and serves as nursery habitats for over 70 invertebrate and fish species (Sea Country Solutions 2023). Seagrass and mixed submerged aquatic vegetation were widespread in the shallow and sandy areas of the lagoon in the 1990s (Smithers 1994; Williams 1994). Buckee et al. (2021) have since estimated that ~80% of seagrass has been lost between 2006 and 2018, likely due to a combination of ocean warming and turbidity, combined with grazing pressure on the remaining seagrass by turtles. Local knowledge holders, confirmed by recent lagoon‐wide surveys (Bell et al. 2025), indicate that the spatial extent of seagrass remains similar across the lagoon but with low coverage (1%–5% cover) compared to the 1990s (10%–75%; Buckee et al. 2021). *Thalassia hemprichii* (Figure 1A) is the dominant seagrass species in the lagoon (Smithers 1994, Williams 1994) and is distributed widely across the Indo West Pacific (Larkum and Kendrick 2018). *Syringodium isoetifolium* is also present in the lagoon and outer reef (Williams 1994) but was not present at the study sites. *Thalassodendron ciliatum* was also historically present across ~80 ha in deeper waters (~8 m) but is now considered locally extinct (Konzewitsch and Evans 2024).

**FIGURE 1:**
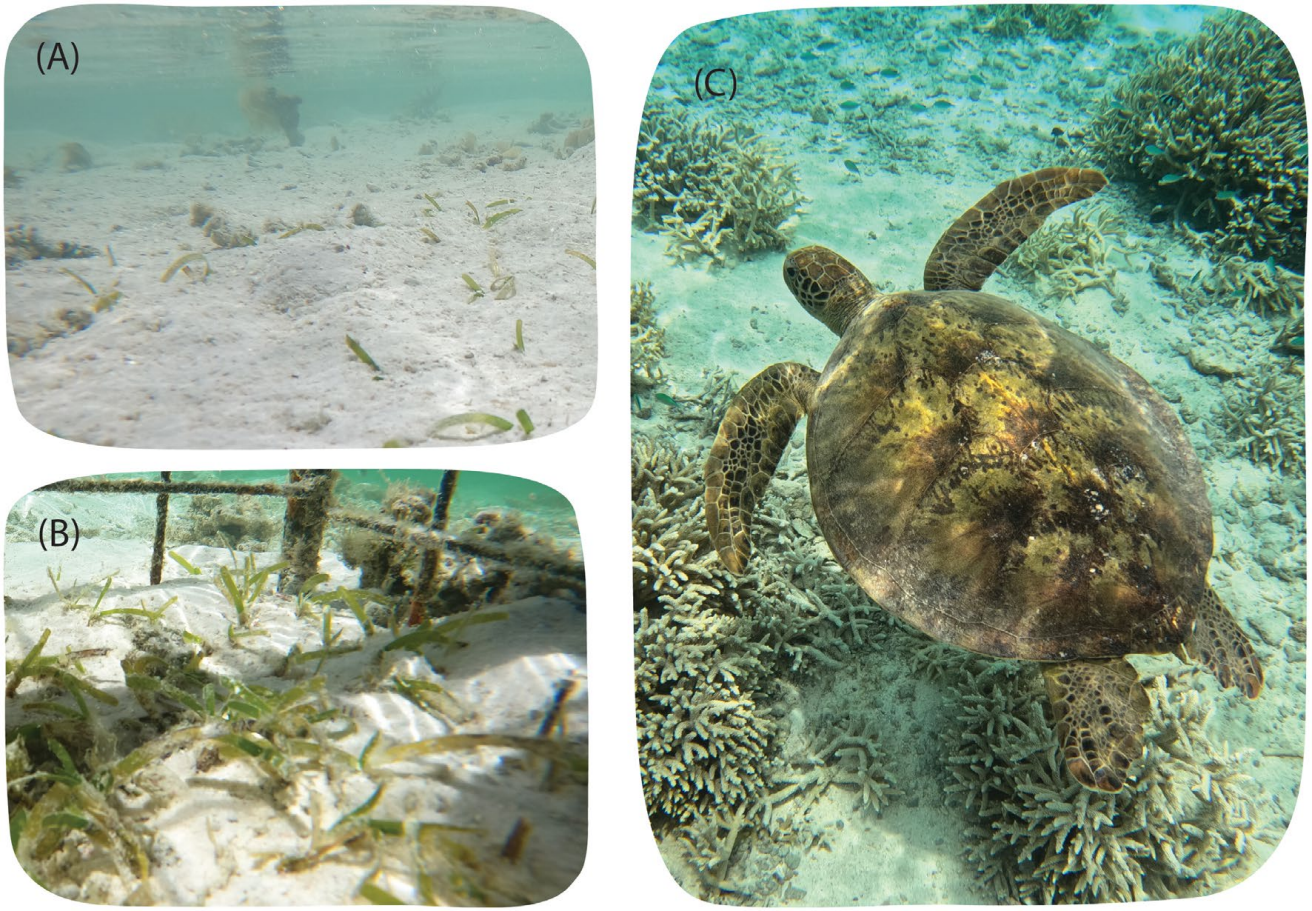
Photographs of the study species *Thalassia hemprichii* in grazed plots (A) and within cages (B), and a green turtle swimming through coral habitat (C).

Seagrass is an important food source for ‘*penyu*’ (green sea turtles or *Chelonia mydas* , Figure 1C), a culturally important and iconic species for the islands and of conservation importance as a genetically distinct population in a global context (Whiting et al. 2014; Dethmers et al. 2010). Hawksbill turtles ( *Eretmochelys imbricata* ) are also present although their diet is dominated by algae and sponge, but can also include seagrass (Whiting et al. 2014). Unlike most turtle populations, which have experienced severe commercial exploitation and sustained declines (Mazaris et al. 2017), the CKI population was subject to subsistence harvest from the time of settlement in the 1820s until the mid‐1980s, after which harvest declined to negligible levels (Macrae and Whiting 2014). The population subsequently recovered to high foraging densities, and long‐term monitoring since 1999 has characterised the populations as one of the highest densities of green turtle foraging aggregations recorded globally (Whiting et al. 2014).

### 2.2 Experiment Design

Exclusion cages were deployed at two sites near Home Island (Pulu Kampong; Figure 2) to assess the effects of excluding turtle herbivory on seagrass traits and function. This was investigated by comparing seagrass protected by cages with seagrass exposed to grazing. The two sites were approximately 300 m apart in the same region of the lagoon, with similar water conditions depths (0.1–1.3 m), and similar seagrass ( *T. hemprichii* ) shoot density (23.1 ± 2.4 SE) at the start of the experiment. The locations of these sites were determined through consultation with the local community and cages were installed in November 2021 (Bell et al. 2025, Bell et al. unpublished manuscript). There were 4 caged treatment plots (‘caged’) and 4 randomly allocated plots of equal size within the naturally grazed meadow (‘grazed’) at each of the sites (Figure 2). The location of plots was selected randomly within habitats containing mostly sand and in an approximately 15 × 15 m area that defined each site. A distance of at least 3 m was measured between each plot to ensure physiological independence (Marbà et al. 2002). Further, the rhizomes around the edges of the plots were severed with a shovel around each plot following installation of the cages. The cages (0.45 × 0.45 × 0.3 m) were constructed with galvanised steel mesh (110 × 110 mm aperture) secured into the sediment with steel star pickets and reinforcing bars (0.3 m height). This is a common design used in exclusion studies to prevent herbivore access, whilst minimising reduction in light availability and water flow (Bakker, Pagès, et al. 2016; Rodriguez and Heck 2020). The cages were cleaned of biofouling growth every 2 to 3 months, and there were minimal algae or fouling observed over the duration of the experiment. The cages remained in place and were effective at excluding green sea turtles and large fish for 2 years and 3 months, whilst enabling access by mesograzers and smaller fish.

**FIGURE 2:**
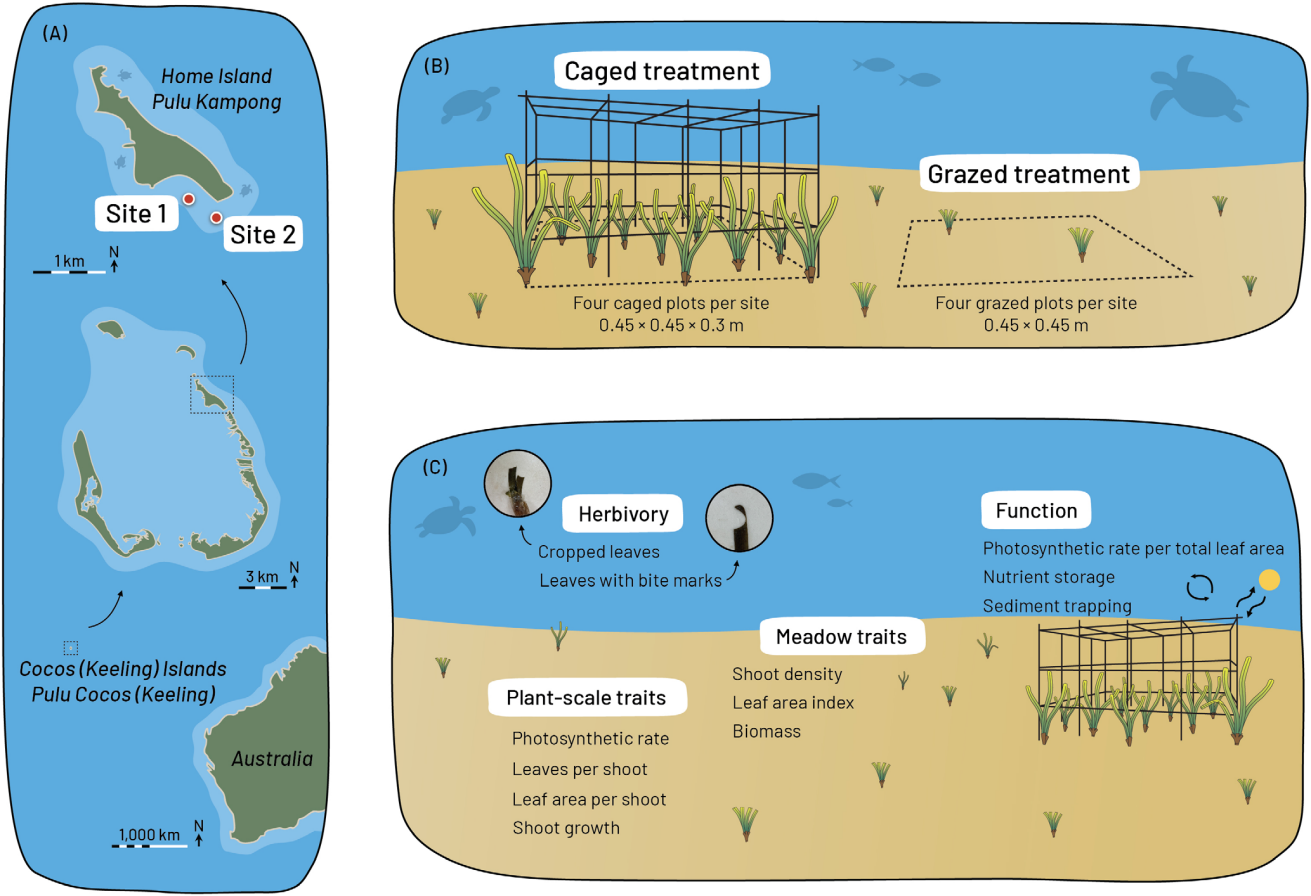
(A) Location of study sites at Home Island (*Pulu Kampong*) at the Cocos (Keeling) Islands relative to Western Australia, (B) schematic diagram of exclusion cages used to prevent turtles and large fish from seagrass for 2 years and 3 months (caged) and plots exposed to natural grazing for the duration of the experiment (grazed) (not to scale), and (C) plant‐scale traits, meadow traits, and function variables compared between treatments and sites.

### 2.3 Data Collection

Following 2 years and 3 months of herbivore exclusion (February 2024), field sampling and harvest of all seagrass material was conducted within the caged and grazed treatments to assess the effect of protection from grazing on plant‐scale and meadow traits, and ecosystem function (Figure 2). Seagrass photophysiology and shoot growth were assessed in the field, prior to collection of sediment and seagrass samples for analysis in the laboratory. Samples were presented to Department of Agriculture, Fisheries and Forestry Biosecurity Officers at the Perth International Airport for inspection and deemed to comply with the Australian Biosecurity Import Conditions. This study was reviewed and approved by the ethics committee at Edith Cowan University (Animal Ethics Committee, project 2024–05747). This project was also reviewed and approved by Cocos Marine Care (a recognised group of representatives of the Cocos (Keeling) Islands community) and permission was granted to collect samples by Parks Australia.

#### 2.3.1 Plant‐Scale Traits

Seven days prior to sample collection, at least 10 shoots inside each caged plot and the surrounding grazed meadows were marked for growth following methods described by Short and Duarte (2001). Despite the same number of shoots marked for shoot growth, not as many samples were recovered in the grazed plots, with only 2–3 shoots recovered per site compared to 2–6 shoots per plot within the caged treatments, most likely due to removal by turtle grazing.

One to two days prior to sample collection, photophysiology was assessed in the field using a pulse‐amplitude modulation (PAM) fluorometer (Diving PAM; Walz, Effeltrich, Germany) on a standardised area (0.2 cm^2^) of young, fully expanded leaves (*n* = 3 per plot). Electron transport rate represents a measure of productivity relative to the absorbed irradiance, and was calculated following Beer and Björk (2000):

$$\begin{array}{c} =\text{effective quantum yield}\;\left(\mathrm{EQY}\right)\times \text{underwater irradiance}\\ \;\times \text{absorption factor}\;\left(\mathrm{AF}\right)\times 0.5 \end{array}$$

The effective quantum yield (EQY) was calculated by placing a leaf exposed to ambient light between the sampler's finger and the fibre optic cable and applying a saturating pulse of light (0.8 s duration) as the ratio of variable fluorescence (${F_{M}}^{\prime }-F^{\prime }$) to maximal fluorescence (${F_{M}}^{\prime }$) following Beer and Björk (2000). The underwater irradiance was measured by placing the sensor at the height of the seagrass leaves facing the water surface (measure 1). The leaf absorption factor (AF) was determined by placing a leaf over the light sensor (measure 2), and calculated as a proportion:

$$=\frac{\text{measure}\;1-\text{measure}\;2}{\text{measure}\;1}$$

The above parameters were multiplied by 0.5 as this represents the fraction of photons absorbed by PSII in plants, and data is expressed in units of micromole electrons per square meter per second (μmol·m^−2^·s^−1^). Measurements were conducted between 9:00 and 11:00 to standardise the light received by the leaves as much as possible and to account for the diurnal cycle of photosynthesis. Due to these time constraints as well as equipment malfunction, one caged plot was not assessed at Site 1 and for 2 plots, 1 at each site, only 2 rather than 3 leaves were measured.

All seagrass material contained within the plots, including shoots marked for growth, was collected by hand and placed into calico bags. The samples were transported to a temporary field laboratory, where they were rinsed in freshwater to remove sediment. The seagrass and sediment samples were stored at −3°C before being transported to laboratory facilities at Edith Cowan University and stored at −18°C until processing. During transit (approximately 8 h by commercial aircraft), the frozen samples were stored with ice bricks in a sealed polystyrene box.

The shoots marked for growth measurements were recovered from the collected sample and processed following methods described by Short and Duarte (2001). Ten shoots were selected randomly to estimate the leaf surface area per shoot (cm^2^ shoot^−1^) by measuring the length and width of each leaf in a shoot using manual callipers. All seagrass material collected from each plot was processed to derive the number of leaves per shoot.

#### 2.3.2 Meadow Traits

The total number of shoots per plot was counted in the laboratory. A time series of shoot density and cover over the exclusion period is reported separately in Bell et al. (unpublished manuscript). All collected seagrass leaves were gently cleaned with fingers to remove epiphyte material, then separated into above and belowground components to measure biomass (grams dry weight per plot). The processed samples were dried in an oven at 60°C for at least 48 h and weighed. The leaf area index (LAI) was calculated by multiplying the mean leaf surface area per shoot (m^2^) by the number of shoots per plot and dividing by the plot area (0.2025 m^2^; Enríquez and Pantoja‐Reyes 2005).

#### 2.3.3 Function

To assess photosynthetic capacity as an ecosystem function, the mean photosynthetic rate measured per standard leaf area in the field prior to sample collection was calculated for each treatment and site combination, then scaled to the total leaf area per plot (m^2^ plot^−1^) using the following formula:

$$=\text{mean}\;\mathrm{ETR}\times \left(\text{leaf area}\;m^{2}{\text{shoot}}^{-1}\times \text{shoot count}\right)$$

To assess nutrient storage capacity, one subsample (~0.5 g dry weight) of leaf and one subsample (~0.5 g dry weight) of rhizome material were finely ground using a ball mill grinder to analyse for nutrient content (i.e., carbon, nitrogen and phosphorus) per plot. Total carbon (TC) and total nitrogen (TN) were analysed by the Australian National University using an elemental analyser (CEInstruments, Milan, Italy, EA1110) coupled to a continuous‐flow isotope ratio mass spectrometer (EA‐CF‐IRMS, Micromass Isochrom). Analytical precision based on duplicate analysis was ±1.1% for nitrogen and ±0.5% for carbon (relative percent difference, % dry weight). One subsample of leaf and one subsample of rhizome material was analysed for total phosphorus (TP) at Edith Cowan University by acid digestion (HNO_3_/H_2_O_2_) followed by Inductively Coupled Plasma‐Optical Emission Spectroscopy (iCAP 7600 duo ICP‐OES). Analytical precision for phosphorus based on duplicate samples was ±3.7% (relative percent difference, % dry weight). Nutrient content data were scaled to the biomass per plot by multiplying the percent nutrient by relevant biomass component to derive nutrient storage capacity.

Two replicate cores of surface sediment (depth of 55 mm) were collected prior to seagrass sample collection using a 70 mL container (44 mm diameter and 55 mm length) from within each plot. These two replicate sediment samples from within each plot were pooled, dried in an oven at 60°C, and weighed to derive sediment trapping capacity. Samples were sieved using a 2.8 mm sieve, and one subsample from sieved material was selected per plot to analyse sediment grain size. The subsample was analysed via laser diffraction using a Malvern Mastersizer 3000 particle size analyser with a lens range of 0.01–2800 μm (refractive index: 1.52, samples ultrasonically dispersed at 100% power for 60 s prior to measurement) to yield grain size distribution (McCave et al. 1986). The ISO standard for Laser Diffraction (13,320:2020) was used to confirm the samples were appropriate. Five measurements were completed per plot, and the average of the five measurements was used for subsequent analysis.

Sediment trapping was also assessed by estimating the organic matter content in surface sediment samples by loss on ignition (LOI). One subsample of sediment for each plot was dried in an oven at 105°C for at least 48 h, then placed into pre‐burnt and pre‐weighed crucibles, and weighed to contain between 6.1 and 7.1 g. The samples were placed in a furnace at 550°C for four hours. Once cooled, the crucibles containing the samples were weighed again, and the organic content was calculated by subtracting the post‐burn crucible weight from the pre‐burn weight. The equation used follows Heiri et al. (2001):

$$\mathrm{LOI}=\left(\frac{{\mathrm{DW}}_{105}-{\mathrm{DW}}_{550}}{{\mathrm{DW}}_{105}}\right)*100$$

where ${\mathrm{DW}}_{105}$ represents the dry weight (grams) of the sample before combustion, and ${\mathrm{DW}}_{550}$ the dry weight (grams) of the sample after combusting to 550°C (Heiri et al. 2001).

#### 2.3.4 Herbivory

While herbivory rates were not measured directly across the study, herbivory marks at the time of sample collection were used as an indicator of the effectiveness of the cages for reducing turtle herbivory pressure. All collected seagrass material was inspected for evidence of cropping, typically used to indicate green turtle feeding behaviour of eating the top portion of seagrass leaves (Bjorndal 1980; Figure 2). This was measured by counting the number of leaves in each sample that were cropped. In addition, there are several herbivorous fish species at the CKI, including a few species of parrotfish and rabbitfish (Hobbs et al. 2014), that are reported to graze upon seagrasses. Fish larger than the mesh size were simultaneously excluded by the cages, but it is possible that smaller individuals were able to access the seagrass material within the cages; therefore, all leaves within the samples were also inspected for evidence of circular‐shaped bite marks, typically used as an indicator of fish grazing (Kirsch et al. 2002, Figure 2).

### 2.4 Data Analysis

Statistical analyses were carried out in R (version 4.4.0 Puppy Cup; R Core Team 2024) using the car package (Fox and Weisberg 2019) and vegan package (Oksanen et al. 2025). To test for the interactive effect of turtle exclusion treatment and site on the response variables described above, two‐way analysis of variance (ANOVA) analyses were conducted. When multiple measures were made within a plot (i.e., electron transport rate and leaf growth rate), the mean value was used in statistical analyses. Data were tested for normality using the Shapiro–Wilk test and for homoscedasticity using Levene's test. Significant interactions were explored using Tukey's HSD post hoc tests and simple effects analysis. Data transformations were applied when assumptions were violated: square root transformation for leaf area per shoot, shoot count, and rhizome carbon storage; log+0.001 transformation for LAI, aboveground and belowground biomass, ETR by total leaf area, leaf and rhizome phosphorus storage, and leaf and rhizome nitrogen storage; and square transformation for leaf nitrogen and carbon content. Proportion data with zero‐inflation (i.e., proportion of leaves with bite marks) did not meet assumptions after transformation was analysed using a Mann–Whitney *U* test instead of two‐way ANOVA. Statistical significance for all analyses was set at *α* = 0.05, unless stated otherwise.

Shoot growth (grams dry weight per shoot per day) was analysed with site and treatment as fixed factors; however, the design was unbalanced as not all tagged shoots were recovered from the adjacent grazed area. Within caged plots, multiple shoots were recovered, so the mean growth rate per plot was calculated based on the 2–6 shoots recovered per caged treatment (*n* = 4 cages per site). Very few shoots were recovered from the grazed treatments, so each recovered shoot represented an independent observation and individual measurements were retained (*n* = 5 total; 2 shoots at Site 1 and 3 shoots at Site 2). The unbalanced data were fitted using generalised least squares (gls, nlme package; Pinheiro, Bates, and R Core Team 2026) with maximum likelihood estimation. Marginal (Type III) tests were used and significance was assessed at *α* = 0.05.

To test for differences in the sediment grain size distributions between treatments and sites, a permutational multivariate analysis of variance (PERMANOVA; Anderson 2001) was performed on Euclidean distances with 9999 permutations. The raw laser diffraction data were aggregated into five size classes (0–63, 63–125, 125–250, 250–500, and > 500 μm) representing the proportion of sediment volume in each class. The homogeneity of multivariate dispersions was tested using the betadisper test (Anderson 2006) to validate the PERMANOVA assumptions. Where significant differences were detected, to identify which size classes contributed to compositional differences, independent *t*‐tests were performed for each size class with false discovery rate (FDR) correction using the Benjamini‐Hochberge method to account for multiple comparisons (Benjamini and Hochberg 1995). Degrees of freedom were adjusted using Welch's correction for unequal variances.

## 3 Results

### 3.1 Plant‐Scale Traits

Seagrass inside the cages had more than double the leaf area per shoot compared to plants exposed to grazing (F_1,12_ = 47.12, *p* < 0.001; Figure 3C; Table S1). The treatment effect was clear even with significant differences between sites (F_1,12_ = 5.71, *p* < 0.05; Table S1), with more leaf area at Site 1 than Site 2. Despite differences in leaf area between treatments, the mean number of leaves per shoot were similar, at approximately 3 leaves per shoot and the effect of treatment was not significant (F_1,12_ = 3.85, *p* = 0.07; Figure 3B) as well as sites (F_1,12_ = 0.12, *p* = 0.73; Table S1). Similarly, photosynthetic rate was not affected by treatment, with similar rates (201.50 ± 18.25 μmol e^−^ m^−2^ s^−1^) across treatment (F_1,11_ = 0.78, *p* = 0.40; Figure 3A) and site (F_1,11_ = 0.53, *p* = 0.48; Table S1). There was also no significant difference in shoot growth rate between the grazed and caged treatments (0.62 ± 0.06 mg DW shoot^−1^·day^−1^, F_1,9_ = 1.16, *p* = 0.31; Figure 3D), but there were significant differences between sites (F_1,9_ = 8.63, *p* < 0.05; Table S1) where the growth of plants at Site 1 was almost double Site 2 (0.79 ± 0.09 vs. 0.45 ± 0.08 mg DW shoot^−1^ day^−1^). There was no significant interaction between treatment and site for all variables assessed at the plant‐scale (Table S1).

**FIGURE 3:**
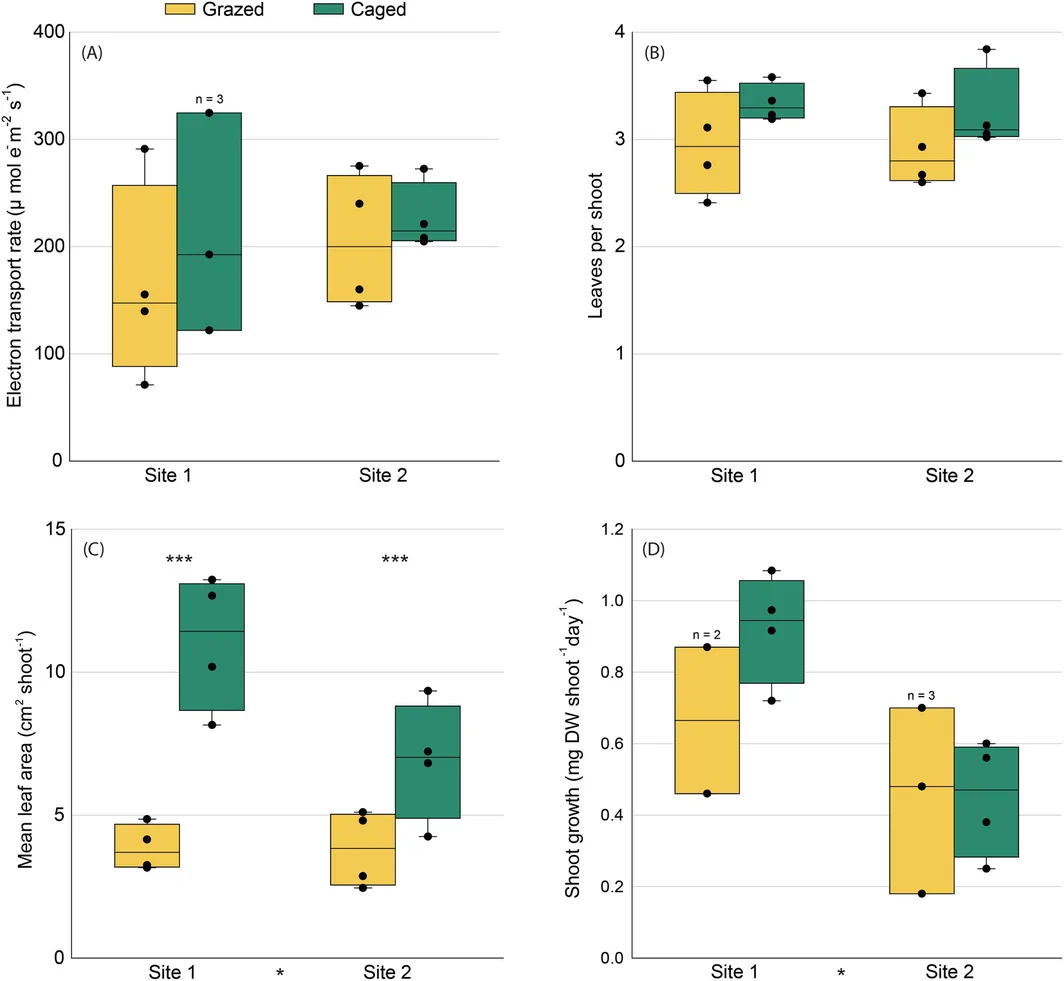
Plant‐scale traits. (A) Electron transport rate (μmol e^−^b7m^−2^·s^−1^), (B) number of leaves per shoot, (C) leaf area per shoot (cm^2^ shoot^−1^) and (D) shoot growth rate (mg DW shoot^−1^·day^−1^) of caged seagrass (green) following 2 years and 3 months of protection from herbivory and seagrass exposed to natural grazing (yellow) at two sites. Lower and upper box boundaries are the 25th and 75th percentiles, respectively, and the lower and upper error bars are the 10th and 90th percentiles, respectively. The circles are the raw data for each replicate plot (*n* = 4, except indicated otherwise), and the horizontal line is the median. Asterix above the boxes indicate statistical significance for treatment effect and site effect is indicated on the x‐axis (*p < 0.05, **p < 0.01, and ***p < 0.001).

### 3.2 Meadow Traits

Caged plots had almost 4 times more shoots than plots exposed to grazing (93.9 ± 19.1 vs. 24.0 ± 5.5; F_1,12_ = 24.4, *p* < 0.001; Figure 4A; Table S1). There were also more than double the shoots at Site 1 than at Site 2 (82.4 ± 19.4 vs. 35.5 ± 14.5; F_1,12_ = 12.60, *p* < 0.01), however there was no interaction between treatment and site (F_1,12_ = 0.86, *p* = 0.37; Table S1). The leaf area index (LAI) was 9 times higher in caged plots compared to grazed plots (0.46 ± 0.12 LAI vs. 0.05 ± 0.01 LAI; F_1,12_ = 45.35, *p* < 0.001; Figure 4B; Table S1). The LAI at Site 1 was more than triple that of Site 2 (0.39 ± 0.14 vs. 0.12 ± 0.06 LAI; F_1,12_ = 16.05, *p* < 0.01), and no interaction (F_1,12_ = 0.37, *p* = 0.55; Table S1). Caged treatments had approximately 7‐fold more aboveground biomass (F_1,12_ = 30.20, *p* < 0.001; Figure 4D) and 7‐fold more belowground biomass (F_1,12_ = 29.95, *p* < 0.001; Figure 4E; Table S1) compared to the grazed plots. There were also site‐level differences in both aboveground and belowground biomass (both *p* < 0.01; Figure 4D and Figure 4E; Table S1), where there was three times more biomass at Site 1 compared to Site 2. The ratio between aboveground and belowground biomass remained similar between the caged and grazed seagrass (F_1,12_ = 0.43, *p* = 0.001; Figure 4C; Table S1), indicating the plants are proportionally smaller but structurally similar.

**FIGURE 4:**
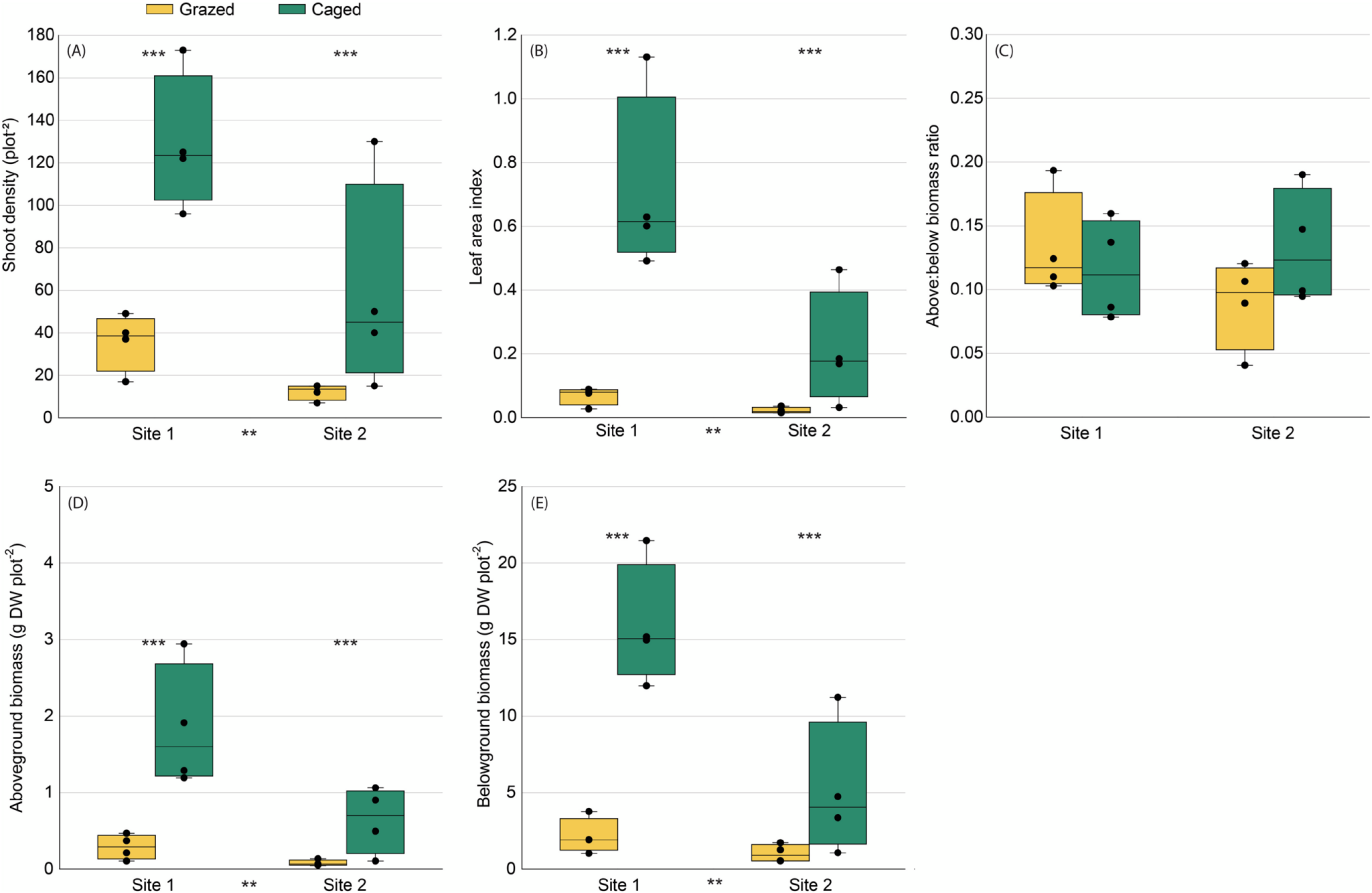
Meadow traits. (A) Shoot density per plot, (B) leaf area index, (C) above: Below biomass ratio, (D) aboveground biomass (g DW plot^−2^), and (E) belowground biomass (g DW plot^−2^) and of caged seagrass (green) following 2 years and 3 months of protection from herbivory and seagrass exposed to natural grazing (yellow) at two sites. Lower and upper box boundaries are the 25th and 75th percentiles, respectively, and the lower and upper error bars are the 10th and 90th percentiles, respectively. The circles are the raw data for each replicate plot (*n* = 4) and the horizontal line is the median. Asterix above the boxes indicate statistical significance for treatment effect and site effect is indicated on the x‐axis (*p < 0.05, **p < 0.01, and ***p < 0.001).

### 3.3 Function

While there was no difference in the photosynthetic rate per standard leaf area at the plant‐scale, there was significantly greater net photosynthetic rates in the caged plots compared to grazed plots when scaled to the total leaf area (19.0 ± 4.74 vs. 1.49 ± 0.31 μmol e^−^·plot^−1^·s^−2^; F_1,12_ = 52.71, *p* < 0.001; Figure 5A; Table S1). There was also an effect of site, with Site 1 being greater than Site 2 where there was more leaf area (15.14 ± 5.59 vs. 5.36 ± 2.54 μmol e^−^·plot^−1^·s^−2^, F_1,12_ = 10.54, *p* < 0.01), but no interaction between treatment and site (F_1,12_ = 0.76, *p* = 0.40; Table S1).

**FIGURE 5:**
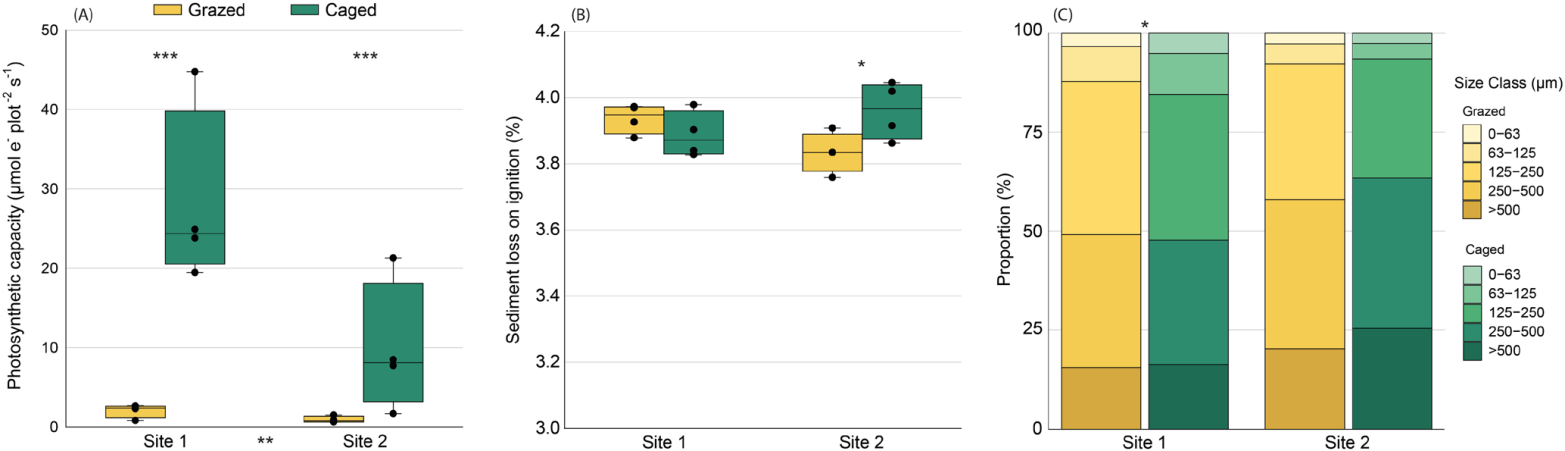
Function. (A) Photosynthetic capacity scaled to leaf area per plot (μmol e^−^·plot^−2^·s^−1^), (B) sediment loss on ignition (%), and (C) sediment grain size proportion (%) of caged seagrass (green) following 2 years and 3 months of protection from herbivory and seagrass exposed to natural grazing (yellow) at two sites. Box plots (A and B) show lower and upper box boundaries with the 25th and 75th percentiles, respectively, and the lower and upper error bars are the 10th and 90th percentiles, respectively. The circles are the raw data for each replicate plot (*n* = 4), and the horizontal line is the median. Asterix above the boxes indicate statistical significance for treatment effect and site effect is indicated on the x‐axis (*p < 0.05, **p < 0.01, and ***p < 0.001). Stacked bars (C) shows proportion of size classes from finest (0–63 μm, lightest colour) to coarsest (> 500 μm, darkest colour) sediment. PERMANOVA analysis detected significant compositional differences between the grazed and caged treatments at Site 1 (*p < 0.05) but not at Site 2 (*p* = 0.34).

There was a significant interaction between treatment and site for the sediment grain size distribution (PERMANOVA: F_3,12_ = 6.52, *p* < 0.01; Figure 5C; Table S2). Site‐specific tests revealed that this was due to treatment differences only at Site 1, explaining 76% of variation in sediment composition (F_1,6_ = 19.07, *p* < 0.05). Subsequent independent *t*‐tests for Site 1 indicated there was higher silt‐clay (< 63 μm) and very fine sand (63–125 μm) within the cages compared to the grazed plots (*p* < 0.01 and *p* < 0.05, respectively; Table S3). In contrast, there was higher fine sand and medium sand within the grazed plots compared to the cages (*p* < 0.05; Table S3). There were no significant differences at Site 2 (F_1,6_ = 1.37, *p* = 0.34).

There was also a significant interaction for sediment organic matter content (F_1,12_ = 6.91, *p* < 0.05; Figure 5B). Post hoc pairwise comparisons revealed that the caged plots at Site 2 had significantly higher organic matter content (3.96% ± 0.03%) than the grazed plots (3.83% ± 0.03%; *p* < 0.05; Table S1), although the effect size was small (0.1%). In contrast, there was no significant difference at Site 1 (caged: 3.89% ± 0.03% vs. grazed: 3.94% ± 0.03%; *p* = 0.32; Table S1).

Nutrient content in leaves ranged from 1.16% to 3.70% nitrogen (N), 29.4% to 39.2% carbon (C), and 0.18% to 0.44% phosphorus (P), while rhizomes ranged from 0.94% to 1.42% N, 28.1% to 37.7% C, and 0.06% to 0.25% P. Stoichiometric ratios ranged from 11.8 to 37.3 (C:N) and 216.3 to 543.0 (C:P) in leaves, and 27.2 to 40.2 (C:N) and 371.0 to 1574.5 (C:P) in rhizomes. When scaled to the relevant biomass component, there was 6‐fold more nitrogen (F_1,12_ = 20.53, *p* < 0.001), 7‐fold carbon (F_1,12_ = 19.96, *p* < 0.001), and 6‐fold more phosphorus (F_1,12_ = 23.10, *p* < 0.001; Figure 6; Table S1) stored in the leaf material of cages compared to the grazed plots. This pattern was similar for rhizome tissues, where caged seagrass had 8 times more nitrogen (F_1,12_ = 28.25, *p* < 0.001) and 9 times more phosphorus (F_1,12_ = 33.50, *p* < 0.001; Figure 6; Table S1) compared to the grazed treatments. There was also a significant effect of site on leaf tissues, where Site 1 stored almost 3 times more nitrogen (F_1,12_ = 9.14, *p* < 0.01) and carbon (F_1,12_ = 8.36, *p* < 0.05), as well as almost 4 times more phosphorus (F_1,12_ = 10.98, *p* < 0.01; Figure 6; Table S1) compared to Site 2. There was no significant site effect on phosphorus stored in rhizome tissues (F_1,12_ = 4.60, *p* = 0.053). There was a significant interaction between treatment and site for carbon stored in rhizome tissues, with simple effect analysis indicating that treatment had a stronger effect at Site 1 (10‐fold reduction from caged to grazed; F_1,12_ = 72.38, *p* < 0.001; Table S1), with a similar but weaker trend at Site 2 (6.4‐fold reduction; F_1,12_ = 5.86, *p* = 0.052; Table S1). All other stored nutrients had no significant interaction (all *p* > 0.05; Table S1).

**FIGURE 6:**
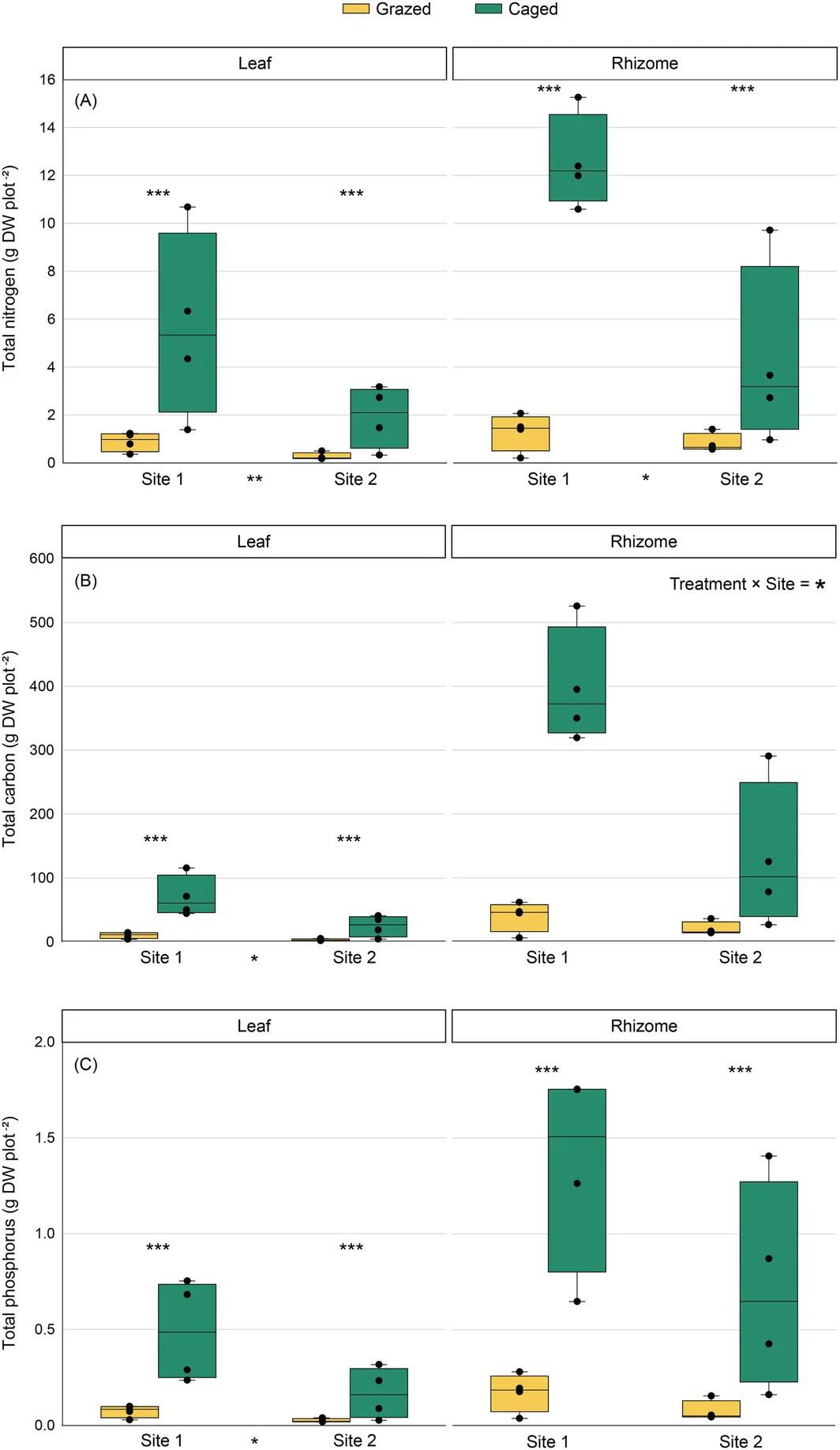
Function. (A) Nitrogen, (B) carbon and (C) phosphorus (g DW plot^−2^) stored in leaves and rhizomes of caged seagrass (green) following 2 years and 3 months of protection from herbivory and seagrass exposed to natural grazing (yellow) at two sites. Lower and upper box boundaries are the 25th and 75th percentiles, respectively, and the lower and upper error bars are the 10th and 90th percentiles, respectively. The circles are the raw data for each replicate plot (*n* = 4), and the horizontal line is the median. Asterix above the boxes indicate statistical significance for treatment effect and site effect is indicated on the x‐axis (*p < 0.05, **p < 0.01, and ***p < 0.001).

### 3.4 Herbivory

Seagrass inside the cages had half the number of leaves cropped (mean 26.2% ± 3.9% standard error) compared to the grazed treatments (58.1% ± 3.1%; F_1,12_ = 45.0, *p* < 0.001; Figure S1A; Table S1), and this was consistent at both sites (F_1,12_ = 0.36, *p* = 0.56). Semicircular bites in the leaves were rarely observed and occurred only in caged treatments, and on between 2.1% and 3.3% of leaves (Figure S1B), with no significant differences between treatments and sites (Mann–Whitney U: W = 44, *p* = 0.08 and W = 28, *p* = 0.59; respectively; Table S1).

## 4 Discussion

Despite ongoing turtle herbivory that limits meadow state and functionality, our results demonstrate that *T. hemprichii* retains some capacity to recover following reduction in grazing pressure for more than two years (Table 1). The magnitude of difference in shoot density and aboveground biomass within cages translated to greater net photosynthetic performance, suggesting that temporary relief from herbivory can restore important ecosystem services like primary production (Scott et al. 2018). Continued exposure to herbivory led to decreased leaf area, lower shoot counts, and reduced biomass (Table 1), which corroborates similar studies that have found comparable effects (Fourqurean et al. 2010; Gulick et al. 2020; Scott et al. 2020; Putillo‐Wehry et al. 2025). At the functional scale of photosynthesis (based on fluorometry) when scaled to total leaf area, the overall photosynthetic rate and subsequent productivity were substantially lower in grazed plots. However, responses varied across the two study sites and several other plant‐scale traits remained unaffected (Table 1). These findings indicate that the ecological services that seagrass provide are compromised at the CKI under current grazing regimes.

**TABLE 1:** Summary of two‐way ANOVA results for how herbivory treatment and site affect the plant‐scale and meadow traits, as well as function.

Scale	Variable	Herbivory treatment	Site				Interaction
Herbivory	Cropped leaves	Caged < Grazed***	Site 1 = Site 2^ns^				
	Bitten leaves	Caged = Grazed^ns^	Site 1 = Site 2^ns^				
Plant	Electron transport rate	Caged = Grazed^ns^	Site 1 = Site 2^ns^				
	Leaves per shoot	Caged = Grazed^ns^	Site 1 = Site 2^ns^				
	Leaf area per shoot	Caged > Grazed***	Site 1 > Site 2*				
	Shoot growth per shoot	Caged = Grazed^ns^	Site 1 > Site 2*				
Meadow	Shoot count	Caged > Grazed***	Site 1 > Site 2**				
	Leaf area index	Caged > Grazed***	Site 1 > Site 2**				
	Aboveground biomass	Caged > Grazed***	Site 1 > Site 2**				
	Belowground biomass	Caged > Grazed***	Site 1 > Site 2**				
	Above: below ratio	Caged = Grazed^ns^	Site 1 = Site 2^ns^				
Function	Photosynthetic capacity	Caged > Grazed***	Site 1 > Site 2*				
	Fine particles (sediment grain size)	Site 1 Caged >^a^ Grazed*					
		Site 2 Caged =^a^ Grazed^ns^					
	Sediment organic matter	Site 1 Caged = Grazed^ns^					*
		Site 2 Caged > Grazed*					
	Leaf total nitrogen storage	Caged > Grazed***	Site 1 > Site 2**				
	Rhizome total nitrogen storage	Caged > Grazed***	Site 1 > Site 2*				
	Leaf total carbon storage	Caged > Grazed***	Site 1 > Site 2*				
	Rhizome total carbon storage	Site 1 Caged > Grazed***					*
		Site 2 Caged = Grazed^ns^					
	Leaf total phosphorus storage	Caged > Grazed***	Site 1 > Site 2**				
	Rhizome total phosphorus storage	Caged > Grazed***	Site 1 = Site 2^ns^				

*Note:* For each significance effect, the symbol indicates which level had the higher mean (e.g., Caged > Grazed denotes a higher mean in caged plots).

^a^ Particle size was significantly different only at Site 1 and no differences were detected at Site 2 based on PERMANOVA analysis.

Asterisks denote the significance of the effect: *p < 0.05, **p < 0.01, and ***p < 0.001, ns = not significant.

Belowground biomass in this study was greatly reduced (85%) by ongoing exposure to herbivory, contrasting to a recent meta‐analysis by Putillo‐Wehry et al. (2025) who found that excluding herbivory generally had minimal effects on belowground biomass. Reduced belowground biomass is unlikely to have resulted from direct consumption by turtles, and may instead reflect the metabolic demands of supporting growth (Fourqurean et al. 2010; Lacey et al. 2014; Martínez López et al. 2019). Although feeding on belowground material has been documented elsewhere (Scott et al. 2020; Christianen et al. 2014) and anecdotally reported in the CKI (Buckee et al. 2021), the direct consumption of *Thalassia* rhizomes is considered rare (Bjorndal 1980; Gulick, Meylan, et al. 2021) and we did not observe this feeding behaviour in footage captured in subsequent studies (O'Dea et al. unpublished data). Furthermore, the proportional reduction across both above and belowground biomass, demonstrated by similar above to belowground biomass ratios, means that grazed plants are proportional miniatures of caged plants. This pattern suggests that the response to chronic herbivory pressure involves proportional biomass scaling rather than a strategic reallocation of resources, which is consistent with plants operating under severe metabolic constraints (Fourqurean et al. 2010). As caged and grazed plots were established within close spatial proximity and were exposed to similar environmental conditions, the design attempts to isolate the effect of herbivory and not other drivers. Simultaneously, plant condition in both treatments reflects the cumulative influence of all pressures acting on these meadows. Seagrass cover across the lagoon is low (Bell et al. 2025), and while this study does not attempt to diagnose historic factors, turbidity and thermal stress have been implicated by other studies of varied temporal and spatial scope (Buckee et al. 2021; Konzewitsch and Evans 2024). Contemporary turtle herbivory is therefore superimposed on a system already in a reduced state, irrespective of the drivers responsible. The combination of high turtle densities (Macrae and Whiting 2014; Whiting et al. 2014), presently low seagrass cover (Bell et al. 2025), and persistent life‐history characteristics of *Thalassia* (Kilminster et al. 2015) in the CKI may be driving a dynamic where seagrasses are maintained in a chronically resource‐depleted state under the existing herbivory pressure. If grazing pressure were to intensify beyond current levels, or coincide with periods of thermal stress or elevated turbidity, the system might approach a threshold beyond which shoots or plants become physiologically compromised with the potential for ecological collapse (Christianen et al. 2021). Compromised resistance capacity could be indicated by shifts in allocation strategies toward survival instead of growth or reduced shoot growth rates, both of which could serve as indicators of imminent tipping point (Monaco and Helmuth 2011; O'Brien et al. 2018).

The effect of turtle exclusion on sediment ecosystem functions, including sediment trapping and organic carbon storage was more complex and site‐specific compared to other variables. There was greater trapping capacity of fine particles in caged seagrass at Site 1, likely driven by greater seagrass abundance and biomass at this site, which would enhance the capacity to attenuate water flow and retain fine particles (Peterson et al. 2004; Hendriks et al. 2010). Seagrass was less abundant at Site 2, particularly belowground biomass, and appeared to have less capacity to trap fine particles. Belowground biomass has a strong influence on sediment stability independently of the canopy (Christianen et al. 2013; Potouroglou et al. 2017), demonstrating the belowground biomass must be retained to provide this important ecological function. Furthermore, the pattern for sediment organic matter appeared to depend more on the hydrodynamic context, rather than exposure to herbivory alone. At the more sheltered Site 1, the organic matter content was similar between both treatments, suggesting that organic matter accumulated in a similar way regardless of seagrass abundance. In contrast, Site 2 is located closer to the inter‐island channel and more exposed to water movement, and here the grazed treatment had significantly lower organic matter than inside the cages. This suggests that seagrass played a more important role in retaining organic matter in an exposed location, likely by attenuating flow velocities and reducing resuspension of organic particles (Hendriks et al. 2008; Samper‐Villarreal et al. 2016). These findings indicate that retention of belowground biomass along with favourable local conditions mediate the extent to which seagrass influences sediment properties, with the trapping function being more effective with maintained belowground biomass (Christianen et al. 2013, Potouroglou et al. 2017), as well as more critical under greater wave exposure (Fonseca and Bell 1998; Koch et al. 2006). These results can inform decision on long‐term management of these systems, including how sites are selected for interventions such as restoration and how monitoring variables are selected.

Dramatic reductions in the nutrient storage capacity documented in this study highlight the implications of reduced seagrass biomass. Seagrasses are widely recognised as important nutrient sinks in coastal and lagoon environments (Touchette and Burkholder 2000), which can also support the food web in the ecosystem (Duffy 2006; Coll et al. 2011). A reduction of seagrass biomass represents a loss of this important function (Aoki and McGlathery 2018; Olivé et al. 2022). Whilst in some instances, herbivory can alleviate the negative effects of eutrophication through the stimulation of seagrass production (Christianen et al. 2012), excessive nutrients or intensive herbivory can shift this balance (Hays 2005). Low‐biomass meadows can be less resilient to nutrient enrichment, creating a compounding risk whereby meadows that are severely impacted by herbivory are more vulnerable to eutrophication‐driven decline (Gladstone‐Gallagher et al. 2018). Lagoons are particularly vulnerable to nutrient loading, and the historic loss of seagrass represents a reduced capacity for nutrients to be assimilated and retained (McGlathery et al. 2007). In the context of the infrastructure development at the study site, this vulnerability is pertinent as airport runway upgrades and associated infrastructure will increase the human population and associated wastewater treatment (Department of Infrastructure, Transport, Regional Development and Communities 2022). Increased human occupation can elevate nutrient inputs to adjacent lagoons (Lapointe et al. 2004; Reopanichkul et al. 2010; Kegler et al. 2018), therefore, this represents a risk to increased nutrient loading to the lagoon ecosystem. Potential sources of nutrients should be carefully monitored and managed, as under current conditions it is unlikely that seagrass at the CKI have capacity to buffer these inputs.

Beyond the biogeochemical implications, loss of seagrass under ongoing herbivory also has consequences for the trophic interactions within the ecosystem. Reduced seagrass biomass can affect multiple trophic pathways (Heck et al. 2008; Valentine and Heck 2021; Cui et al. 2021). Firstly, direct consumption by herbivorous fish such as some species of parrotfish (i.e., *Leptoscarus vaigiensis* ) or *ikan menyirat* (rabbitfish, *Siganus* spp.) depend on seagrass tissue as a food source (Gullström et al. 2011; Vanderklift et al. 2021) and can change as a result (Inoue et al. 2021). Secondly, seagrasses provide nursery and foraging habitat for species like *gerita* (octopus, *Octopus cyanea* ) and *kakap les kuning* (orange‐striped emperor, *Lethrinus obsoletus* ) (Sea Country Solutions 2023), which in turn support higher trophic levels as prey for larger fish or for human consumption (Jackson et al. 2001; Gillanders et al. 2003; Sheaves et al. 2015). Third, herbivory can disrupt or restructure energy flows. Decomposing seagrass material support detrital pathways for crustaceans, worms and bacteria, which in turn are consumed by fish and invertebrates, linking seagrass to the broader food web (Harrison 1989; Hyndes et al. 2014). Reduced seagrass abundance can disrupt this detrital pathway and consequently, other species that depend on detritivores (Klumpp and Kwak 2005). Turtle herbivory can also reroute this pathway through direct consumption of leaves and channelling primary production through the gut and returning it as finely fragmented, nitrogen‐rich faecal matter instead of decomposing leaf litter, effectively short‐circuiting the detrital cycle (Thayer et al. 1982). Reported reductions in fishing catch (Bell, Weeks, Keegan, et al. 2024) are therefore consistent not only with direct habitat loss, but also with the restructuring of energy flow through multiple trophic pathways.

In contrast to meadow traits and functional attributes, there were several plant‐scale attributes that appeared unaffected. While grazing inherently removes photosynthetic tissue (Strauss and Agrawal 1999), the photosynthetic rate of grazed plants did not appear inhibited by herbivory. Grazed seagrasses maintained photosynthetic performance comparable with caged plants, which could be the mechanism supporting growth once herbivory exclusion measures were introduced (Christianen et al. 2012; Gulick et al. 2020; Marín‐Guirao et al. 2022). Data on photosynthetic and growth rates immediately following exclusionary measures were not recorded in this study and would be informative to explore the potential for compensatory responses in future work. While grazed plants retained the physiological capacity to continue growing even under ongoing herbivory pressure, the growth rates recorded in this study (0.62 ± 0.06 mg shoot^−1^·day^−1^) were lower than those reported for *T. hemprichii* elsewhere: 0.76 to 5.90 mg shoot^−1^·day^−1^ (Brouns 1985; Taylor et al. 2011; Liu et al. 2022). This pattern was consistent when scaled to areal basis (g m^−2^·day^−1^), with values in this study remaining substantially lower than growth rates reported for *Thalassia* across the Tropical Indo‐Pacific and Tropical Atlantic bioregions (Strydom et al. 2023). Whether the comparatively low growth rates observed here reflect a characteristic feature of *T. hemprichii* at the CKI remain unknown, representing a significant gap in our understanding of this remote ecosystem.

It is possible that the treatments in this study represent two points along a gradient of herbivory intensity, rather than a binary contrast (Christianen et al. 2021). While the exclusion cages substantially reduced herbivory pressure, evidence of grazing at the time of sample collection was still observed. Small or juvenile green sea turtles, with reported head widths of 61–139 mm (Bolten 1999; Palaniappan 2017; Álvarez‐Varas et al. 2022), were likely capable of accessing seagrass around the inner perimeter of cages constructed with 110 × 110 mm mesh. This behaviour has since been confirmed through video footage in subsequent experiments (O'Dea et al. unpublished data). The caged plots in this study represent a state in which seagrass can accumulate structural complexity and functional capacity with reduced grazing pressure. In contrast, the grazed plots reflect a low‐biomass and structurally simplified condition, more consistent with seagrass condition observed across the lagoon (Bell et al. 2025). Collectively, these observations align with Christianen et al. (2021) in that tropical seagrass meadows can function as spatially and temporarily dynamic systems along a continuum of seagrass abundance and grazing pressure. While the broader ecosystem at our study site is under substantial herbivory pressure, there is potential for seagrass to regain important traits and functions when herbivory is reduced (Figure 7).

**FIGURE 7:**
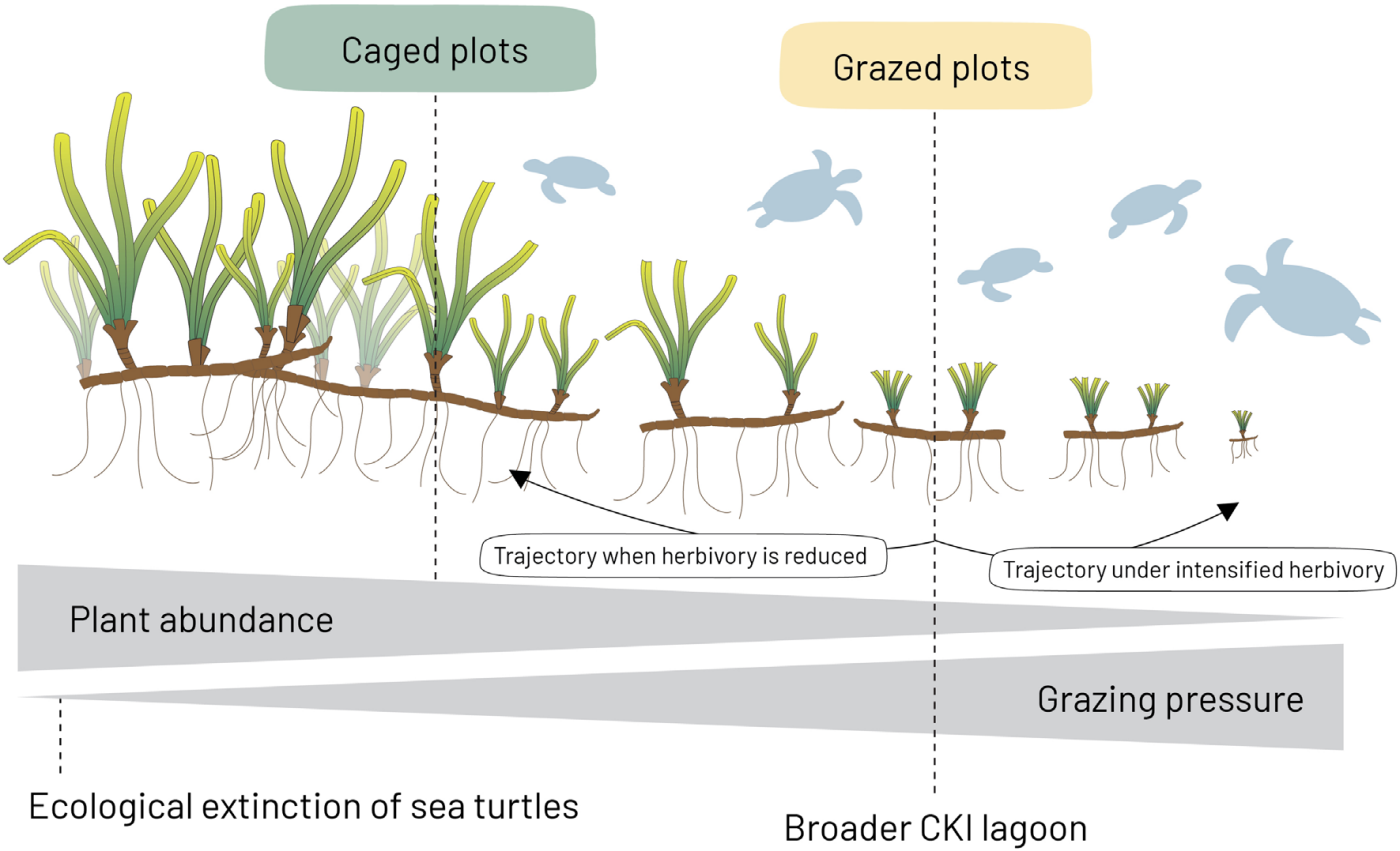
Conceptual diagram illustrating the position of experimental treatments based on differences in shoot density and biomass and the broader Cocos (Keeling) Islands lagoon within a seagrass‐turtle continuum, adapted from Christianen et al. (2021, 2023).

It is also important to acknowledge that cropped leaves, while conventionally attributed to turtle herbivory (Bjorndal 1980; Kelkar et al. 2013), could indicate the presence of other herbivores. Fish typically feed on individual leaves and leave behind crescent‐shaped bites which could produce a cropped appearance when repeatedly biting the same leaf or shoot (Kirsch et al. 2002; Alcoverro and Mariani 2004; Kelkar et al. 2013). Fish herbivory within the grazed plots would have likely been masked by more extensive cropping (Bjorndal 1980; Thayer et al. 1984; Kelkar et al. 2013). Bite marks were detected exclusively within the caged plots, consistent with both masking effects and the possibility that caged plots had greater resources available to observe fish herbivory, attracted herbivorous fish with greater forage availability (Yeager et al. 2011) or offered refuge from predation by larger fish (Doherty and Sale 1986; Gaynor et al. 2019), an acknowledged risk of cage‐based experiment design (Connell 1997).

This study highlights the need for nuanced management strategies that can accommodate the inherently dynamic nature of turtle‐seagrass interactions. Effective management requires regular monitoring of seagrass condition to track further decline or improvement; an objective identified as a priority by marine park managers (Director of National Parks 2025) and the local community (Bell et al. 2024). Targeted interventions such as partial exclusion measures or enhancement of natural physical deterrents may enhance seagrass persistence without compromising green turtle conservation goals. Minimising concurrent anthropogenic stressors on seagrass, such as water quality degradation and coastal development, would support resilience and reduce the risk of compounding effects that could accelerate decline (Valentine and Heck 2021). Furthermore, establishing a carrying capacity of green turtles at the CKI that accounts for the growth dynamics of *T. hemprichii* in the local context would provide useful insight on whether productivity can sustain current grazing pressure, or if the system is at risk of transitioning to a state where seagrass meadows, and their critical ecological, socioeconomic and cultural services, collapse entirely (Rodriguez and Heck 2021).

Reducing herbivory in an ecosystem experiencing grazing pressure had clear benefits to seagrass meadows. While herbivory appeared to be the main driver of differences across these meadow traits and functionality, the location of the cages also appeared to play a role, and the condition of these meadows more broadly is likely to reflect the combined influence of turtle grazing and other co‐occurring pressures. The recovery of green turtle populations is a testament to decades of dedicated conservation effort and stands as one of the rare success stories, yet this is not without consequence for the seagrass ecosystems upon which turtles depend. Local turtle populations, extremely low seagrass cover, and persistent life‐history characteristics of *Thalassia hemprichii* may be driving a dynamic where seagrasses are maintained in a chronically resource‐depleted state with limited capacity for improvement without intervention. Where herbivory is layered over stressors such as elevated temperatures, increased turbidity and coastal development, the resulting metabolic constraints may compromise the capacity of seagrasses to persist (Valentine and Heck 2021) and limit the provision of ecosystem services and contributions to human communities (McKenzie et al. 2021). Adaptive and nuanced approaches to the concurrent management of both turtles and their foraging habitat are needed.

## Author Contributions

**Caitlyn O'Dea:** formal analysis (lead), funding acquisition (equal), investigation (lead), methodology (lead), project administration (lead), resources (lead), visualization (lead), writing – original draft (lead), writing – review and editing (equal). **Kathryn McMahon:** conceptualization (supporting), funding acquisition (supporting), investigation (supporting), methodology (supporting), resources (supporting), supervision (lead), writing – review and editing (equal). **Osman Macrae:** conceptualization (equal), investigation (supporting). **Shakirin Keegan:** conceptualization (equal), investigation (supporting). **Sahira Bell:** conceptualization (equal), funding acquisition (equal), investigation (supporting), methodology (supporting), project administration (supporting), resources (supporting), supervision (supporting), writing – review and editing (supporting).

## Funding

This work was supported by the Department of Education, Australian Government, Research Training Program. Department of Climate Change, Energy, the Environment and Water, Indian Ocean Territories Marine Parks Grants.

## Conflicts of Interest

The authors declare no conflicts of interest.

## Supporting information


Figure S1
Table S1
Table S2
Table S3

## Data Availability

Data files associated with this article will be accessible via Edith Cowan University's Institutional Repository: https://doi.org/10.25958/ywjs‐2093.
