# Supplementary material for: Turtle Herbivory Suppresses Seagrass Function but Reveals Recovery Potential in a Remote Coral Reef Atoll
Source: Ecol Evol. 2026 Oct 24;16(10):e74466. doi: 10.1002/ece3.74466 (PMC13695049; doi:10.1002/ece3.74466)
Supplement: Supplementary file 1 [file ECE3-16-e74466-s001.docx]

Supplementary Figure S1 Proportion of cropped leaves indicative of turtle herbivory (A) and leaves with bite marks indicative of fish herbivory (B) of caged seagrass (green) following 2 years and 3 months of protection from herbivory and seagrass exposed to natural grazing (yellow) at two sites. Lower and upper box boundaries are the 25^th^ and 75^th^ percentiles, respectively, and the lower and upper error bars are the 10^th^ and 90^th^ percentiles, respectively. The circles are the raw data for each replicate plot (n = 4), and the horizontal line is the median. Asterix above the boxes indicate statistical significance for treatment effect.

Supplementary Table S1 Summary of two-way ANOVA results testing for significant effect of treatment (Caged vs Grazed), site (Site 1 vs Site 2) and interaction (treatment × site) on herbivory, plant-scale traits, meadow traits and function. F-values (F), degrees of freedom and residual degrees of freedom (df, rdf) and *p*-value (**p* < 0.05, ***p* < 0.01, ****p* < 0.001, ns = not significant) are shown for all main effects and interactions. Values show the mean ± standard error, where DW = dry weight; above-ground biomass = leaf + living sheath; below-ground biomass = remnant sheaths, rhizome and root material; TN = total nitrogen; TC = total carbon, and TP = total phosphorus.

|  |  | Treatment | | | Site | | | Treatment × Site | | | |  | Treatment (mean ± standard error) | | | | | | Site (mean ± standard error) | | | | | |
| --- | --- | --- | --- | --- | --- | --- | --- | --- | --- | --- | --- | --- | --- | --- | --- | --- | --- | --- | --- | --- | --- | --- | --- | --- |
|  |  | F | df, rdf | *p* | F | df, rdf | *p* | F | df, rdf | *p* | |  | Caged | | | Grazed | | | Site 1 | | | Site 2 | | |
| **Herbivory** | |  |  |  |  |  |  |  |  |  | |  |  |  |  |  |  |  |  |  |  |  |  |  |
|  | Cropped leaves (%) | 45.00 | 1, 12 | *** | 0.36 | 1, 12 | ns | 2.95 | 1, 12 | ns | |  | 26.2 | ± | 3.9 | 58.1 | ± | 3.1 | 43.6 | ± | 5.7 | 40.7 | ± | 8.1 |
|  | Leaves with bites (%) ^[1]^ | W=44 | - | ns | W=28 | - | ns | - | - | - | |  | 0.9 | ± | 0.5 | 0 |  |  | 0.3 | ± | 0.3 | 0.7 | ± | 0.5 |
| **Plant-scale traits** | |  |  |  |  |  |  |  |  |  | |  |  |  |  |  |  |  |  |  |  |  |  |  |
|  | Electron transport rate (μmol e^-^ m^-2^ s^-2^) | 0.78 | 1, 11^[2]^ | ns | 0.53 | 1, 11^2^ | ns | 0.12 | 1, 11^2^ | ns | |  | 220.80 | ± | 24.13 | 184.62 | ± | 26.92 | 185.15 | ± | 34.75 | 215.81 | ± | 16.74 |
|  | Leaves per shoot | 3.85 | 1, 12 | ns | 0.12 | 1, 12 | ns | 0.01 | 1, 12 | ns | |  | 3.30 | ± | 0.10 | 2.93 | ± | 0.14 | 3.15 | ± | 0.14 | 3.08 | ± | 0.14 |
|  | Leaf area (cm^2^ shoot^-1^) ^[3]^ | 47.12 | 1, 12 | ******* | 5.71 | 1, 12 | ***** | 3.75 | 1, 12 | ns | |  | 9.07 | ± | 0.71 | 3.83 | ± | 0.51 | 7.46 | ± | 1.48 | 5.35 | ± | 0.82 |
|  | Shoot growth (mg DW shoot^-1^ day^-1^) | 1.16 | 1, 9^[4]^ | ns | 8.63 | 1, 9^4^ | ***** | 1.27 | 1, 9^4^ | ns | |  | 0.685 | ± | 0.072 | 0.559 | ± | 0.093 | 0.794 | ± | 0.088 | 0.450 | ± | 0.077 |
| **Meadow traits** | |  |  |  |  |  |  |  |  |  | |  |  |  |  |  |  |  |  |  |  |  |  |  |
|  | Shoot count (shoots plot^-1^) ^3^ | 24.40 | 1, 12 | ******* | 12.58 | 1, 12 | ****** | 0.86 | 1, 12 | ns | |  | 93.9 | ± | 19.1 | 24.0 | ± | 5.5 | 82.4 | ± | 19.4 | 35.5 | ± | 14.5 |
|  | Leaf area index ^[5]^ | 45.35 | 1, 12 | ******* | 16.05 | 1, 12 | ****** | 0.37 | 1, 12 | ns | |  | 0.46 | ± | 0.12 | 0.05 | ± | 0.01 | 0.39 | ± | 0.14 | 0.12 | ± | 0.06 |
|  | Above-ground biomass (g DW plot^-1^) ^5^ | 30.20 | 1, 12 | ******* | 13.21 | 1, 12 | ****** | 0.01 | 1, 12 | ns | |  | 1.24 | ± | 0.29 | 0.18 | ± | 0.05 | 1.06 | ± | 0.31 | 0.36 | ± | 0.12 |
|  | Below-ground biomass (g DW plot^-1^) ^5^ | 29.95 | 1, 12 | ******* | 11.88 | 1, 12 | ****** | 1.05 | 1, 12 | ns | |  | 10.50 | ± | 2.21 | 1.59 | ± | 0.35 | 9.03 | ± | 2.31 | 3.06 | ± | 1.21 |
|  | Above:below biomass ratio | 0.43 | 1, 12 | ns | 0.41 | 1, 12 | ns | 2.28 | 1, 12 | ns | |  | 0.12 | ± | 0.01 | 0.11 | ± | 0.01 | 0.12 | ± | 0.01 | 0.11 | ± | 0.01 |
| **Function** | |  |  |  |  |  |  |  |  |  | |  |  |  |  |  |  |  |  |  |  |  |  |  |
|  | Photosynthetic capacity (μmol e^-^ plot^-1^ s^-2^) ^5^ | 52.71 | 1, 12 | ******* | 10.54 | 1, 12 | ****** | 0.76 | 1, 12 | ns | |  | 19.01 | ± | 4.75 | 1.49 | ± | 0.31 | 15.14 | ± | 5.59 | 5.36 | ± | 2.54 |
|  | Sediment organic matter (%) | 1.33 | 1, 12 | ns | 0.20 | 1, 12 | ns | 6.91 | 1, 12 | ***** | Site 1: 3.89 | | | ± | 0.03 | 3.94 | ± | 0.02 |  |  |  |  |  |  |
|  |  |  |  |  |  |  |  |  |  |  | Site 2: 3.96 | | | ± | 0.04 | 3.83 | ± | 0.03 |  |  |  |  |  |  |
|  | Leaf TN storage (g TN DW plot^-2^) ^5^ | 20.53 | 1, 12 | ******* | 9.14 | 1, 12 | ****** | <0.01 | 1, 12 | ns | |  | 3.80 | ± | 1.19 | 0.58 | ± | 0.15 | 3.28 | ± | 1.28 | 1.10 | ± | 0.43 |
|  | Rhizome TN storage (g DW plot^-2^) ^5^ | 28.25 | 1, 12 | ******* | 4.77 | 1, 12 | ***** | 2.37 | 1, 12 | ns | |  | 8.41 | ± | 1.85 | 1.05 | ± | 0.22 | 6.92 | ± | 2.19 | 2.54 | ± | 1.10 |
|  | Leaf TC storage (g DW plot^-2^) | 19.96 | 1, 12 | ******* | 8.36 | 1, 12 | ***** | 4.45 | 1, 12 | ns | |  | 47.28 | ± | 12.04 | 6.43 | ± | 1.80 | 40.07 | ± | 13.64 | 13.64 | ± | 5.59 |
|  | Rhizome TC storage (g DW plot^-2^) ^3^ | 43.85 | 1, 12 | ******* | 12.87 | 1, 12 | ****** | 6.48 | 1, 12 | ***** | Site 1: 397.44 | | | ± | 45.42 | 39.91 | ± | 11.90 |  |  |  |  |  |  |
|  |  |  |  |  |  |  |  |  |  |  | Site 2: 130.17 | | | ± | 57.14 | 20.16 | ± | 5.34 |  |  |  |  |  |  |
|  | Leaf TP storage (g DW plot^-2^) ^5^ | 23.10 | 1, 12 | ******* | 10.98 | 1, 12 | ****** | 0.16 | 1, 12 | ns | |  | 0.33 | ± | 0.09 | 0.05 | ± | 0.01 | 0.28 | ± | 0.10 | 0.09 | ± | 0.04 |
|  | Rhizome TP storage (g DW plot^-2^) ^5^ | 33.50 | 1, 12 | ******* | 4.60 | 1, 12 | ns | 0.02 | 1, 12 | ns | |  | 1.04 | ± | 0.21 | 0.12 | ± | 0.03 | 0.76 | ± | 0.26 | 0.40 | ± | 0.18 |

Supplementary Table S2 Summary of permutational multivariate analysis of variance (PERMANOVA) results testing effect of treatment (Cage vs Grazed) and site (Site 1 vs Site 2) on sediment grain size distribution. Raw laser diffraction data were aggregated into five size classes (0-63, 63-125, 125-250, 250-500, and >500 µm) representing the proportion of sediment volume in each class. Analysis used Euclidean distances with 9,999 permutations. The overall model tested the Treatment × Site interaction across both sites, and site-specific tests examined treatment effects within each site separately. Significance levels: *** = *p* < 0.001, ** = *p* < 0.01, * = *p* < 0.05, and ns = not significant.

| **Analysis** | **Source** | **df** | **Sum of squares** | **F-value** | **p-value** | **Significance** |
| --- | --- | --- | --- | --- | --- | --- |
| Overall model | Model | 3, 12 | 666.41 | 6.52 | 0.0021 | ** |
|  | Residual | 12 | 408.59 |  |  |  |
|  | Total | 15 | 1075.01 |  |  |  |
| Site 1 (site-specific) | Treatment | 1, 6 | 28.65 | 19.07 | 0.0316 | * |
|  | Residual | 6 | 9.01 |  |  |  |
|  | Total | 7 | 37.66 |  |  |  |
| Site 2 (site-specific) | Treatment | 1, 6 | 91.34 | 1.37 | 0.3425 | ns |
|  | Residual | 6 | 399.58 |  |  |  |
|  | Total | 7 | 490.92 |  |  |  |

Supplementary Table S3 Summary of individual size class comparisons at Site 1. Independent t-test (Welch’s t-test for unequal variance) comparing sediment grain size class proportions between caged and grazed treatments. Values are the mean ± standard error (n=4 per treatment). Degrees of freedom (df) were adjusted using Welch’s correction for unequal variances; lower df values for some size classes (63 – 125, 125 – 250 and 250 – 500 μm) reflect variance heterogeneity. The *p*-value was adjusted using the false discovery rate (FDR) method (Benjamini & Hochberg, 1995) to control for multiple comparisons across five size classes. Significance levels: *** = *p* < 0.001, ** = *p* < 0.01, * = *p* < 0.05, and ns = not significant.

| **Particle size** | **Caged** | | | **Grazed** | | | **t** | **df** | **p-value (FDR)** |  |
| --- | --- | --- | --- | --- | --- | --- | --- | --- | --- | --- |
| Silt-clay (< 63 μm) | 5.14 | ± | 0.25 | 3.32 | ± | 0.19 | 5.892 | 5.6 | 0.0065 | ** |
| Very fine sand (63 – 125 μm) | 10.39 | ± | 0.29 | 8.89 | ± | 0.11 | 4.788 | 3.8 | 0.0163 | * |
| Fine sand (125 – 250 μm) | 36.75 | ± | 0.16 | 38.67 | ± | 0.47 | -3.871 | 3.7 | 0.0265 | * |
| Medium sand (250 – 500 μm) | 31.40 | ± | 0.37 | 33.53 | ± | 0.12 | -5.502 | 3.6 | 0.0163 | * |
| Coarse sand (> 500 μm) | 16.34 | ± | 0.23 | 15.58 | ± | 0.33 | 1.867 | 5.4 | 0.1166 | ns |

1. Fish bite data analysed using Mann-Whitney U test due to zero inflation, W statistic reported instead of F-value and interaction not tested for.
2. Electron transport rate and photosynthetic capacity analysis based on n=15 due to equipment failure (i.e., one plot excluded).
3. Raw data transformed before statistical analysis using square root.
4. Analysis performed using generalised linear least squares as not all tagged shoots were recovered from the grazed areas.
5. Data transformed before statistical analysis using log ($x$ + 0.001).
